# Influence of Forced Carbonisation on the Binding Properties of Sludge with a High β-Belite Content

**DOI:** 10.3390/ma14247899

**Published:** 2021-12-20

**Authors:** Aleksandr Bakhtin, Nikolay Lyubomirskiy, Stanisław Fic, Tamara Bakhtina

**Affiliations:** 1 Department of Civil Engineering and Materials Science, Faculty of Architecture and Civil Engineering, Academy of Civil Engineering and Architecture, V.I. Vernadsky Crimean Federal University, Prospekt Vernadskogo 4, 295007 Simferopol, Republic of Crimea; aleserba@gmail.com (A.B.); niklub.ua@gmail.com (N.L.); t.bakhtina83@gmail.com (T.B.); 2 Faculty of Civil Engineering and Architecture, Lublin University of Technology, Nadbystrzycka 40, 20-619 Lublin, Poland

**Keywords:** carbonate hardening, hydration, nepheline sludge, microstructure, mechanical performance, recycling

## Abstract

Alternative binders activated by forced carbonisation are regarded as one of the potential solutions to reducing greenhouse gas emissions, water, and energy consumption. Such binders, in particular those based on nepheline sludge (a by-product of alumina production), cured in carbon dioxide with subsequent hydration, are clinkerless building materials. The development of such binders contributes to the involvement of multi-tonnage solid industrial waste in the production cycle. This type of waste is capable of binding man-made CO_2_ and transforming it into stable insoluble compounds, having binder properties. The optimum technological parameters of the forced carbonisation of the nepheline slime binder was determined by the mathematical planning of the experiment. The novelty of the research is the expansion of the secondary raw material base that can bind the man-made CO_2_ with obtaining the construction products of appropriate quality. It was revealed that the process of active CO_2_ absorption by the minerals of nepheline slime is observed in the first 120 min of the forced carbonization. Immediately after carbonisation, the resulting material develops compressive strength up to 57.64 MPa, and at the subsequent hydration within 28 days this figure increases to 68.71 MPa. Calcium carbonate is the main binder that determines the high mechanical properties of the samples. During the subsequent hydration of the uncoated belite, gel-like products are formed, which additionally harden the carbonised matrix. Thus, after the forced carbonisation and the following 28 days of hardening, the material with compressive strength in the range 4.38–68.71 MPa and flexural strength of 3.1–8.9 MPa was obtained. This material was characterised by water absorption by mass in the range of 13.9–23.3% and the average density of 1640–1886 kg/m^3^. The softening coefficient of the material was 0.51–0.99. The results obtained enables one to consider further prospects for research in this area, in terms of the introduction of additional technological parameters to study the process of forced carbonisation of nepheline slime.

## 1. Introduction

Environmentally friendly construction processes, as well as ensuring a safe and comfortable living environment, are among the most important global trends constituting “sustainable development”, especially in the construction sector. The application of construction materials and elements characterised by low CO_2_ emissions, manufactured based on the technologies that absorb and bind (sequester) anthropogenic carbon dioxide, is the most substantial factor that enhances the environmental friendliness of construction processes. At the same time, the manufacturing of such construction materials and elements based on secondary material resources is an important trend in combating the environmental pollution. Present-day production causes certain problems related to environmental safety, one of which is progressing climate change. According to the publicly expressed international scientific and political opinions, the main reason for this climate change—the rising average annual temperature of the atmosphere–is the aggravation of the greenhouse effect due to increasing amounts of greenhouse gases in the atmosphere. These primarily include carbon dioxide (CO_2_) and methane (CH_4_). According to the data provided by the International Energy Agency (IEA), the annual global emissions of CO_2_ increased from 32 Gt to 37.1 Gt between 2013–2019. Therefore, an ongoing increase in the concentration of carbon dioxide is registered in the atmosphere of the Earth. These data are strongly correlate with the data of the research performed by the scientists from the California Institute of Technology [1]. According to the latter, the concentration of the carbon dioxide equalled 410 ppm at the beginning of 2019. The researchers calculated that if the level of the CO_2_ emission were to remain at the level of 37 Gt annually, then the CO_2_ concentration would increase to 1200 or 1300 ppm by the beginning of the 22nd century. A threefold increase in the carbon dioxide concentration in the atmosphere—up to 1300 ppm–would warm the world’s oceans by 8 °C. The researchers created a climate model showing that at a CO_2_ concentration of over 1000 ppm, the stratocumulus clouds would cease to exist first, followed by the cumulus clouds. One should note that the recovery of the clouds would only become possible by reducing the carbon dioxide concentration by 300 ppm—a level which is 100 ppm lower than it is currently. On a global scale, the consequences of the given scenario are even more negative than those predicted in the International Report [2]. The disclosed concept of the increasing CO_2_ concentration in the atmosphere is confirmed by the analysis of the statistical archive data provided in the paper [3]. The archive data were collected by three various groups of researchers—Remote Sensing Systems (RSS), the Centre for Satellite Applications and Research (STAR), and the University of Alabama at Huntsville (UAH)—from 1979 until 2019. The analysis of the data collected over this 40-year period led to the conclusion that anthropogenic production was 99.9% likely to have caused the global climate change.

The problems of climate change are discussed at the relevant conferences on climate change convened within the framework of UN action. The summary document [4] adopted at the UN Conference on Climate Change held in Madrid in December 2019 stated that some European Union countries (Denmark, Finland, France, Germany, Iceland, Portugal, Spain, and Sweden) have their own national adaptation plans aimed at combating the climate change, according to which they planned to reduce their anthropogenic CO_2_ emissions by 45% by 2030 and reduce the quantity thereof to zero by 2050. In order to achieve the above-mentioned objective, they plan to spend about EUR 900 billion to finance the scientific research looking at how to reduce the CO_2_ emissions in all the possible industrial sectors over the following ten years. That said, the implementation of the results of this scientific research within real sectors of the economy must not negatively impact the economic policy of the European Union and beyond.

It should be noted that the energy, transport, and industrial sectors of the world economy are basic suppliers of anthropogenic CO_2_ to the atmosphere of the Earth. In particular, the production of standard Portland cement and other binding substances alone is responsible for 8% [5,6,7,8] of global anthropogenic CO_2_ emissions and currently amounts to 2.9–3.0 Gt annually. These quantitative indicators of CO_2_ emissions in this specific industrial sector make it a promising candidate in terms of developing technological solutions to reduce and bind (sequester) CO_2_, while simultaneously achieving additional production volume.

The formation and accumulation of millions of tons of solid secondary raw materials in the course of production activities is also a problem. As a rule, this secondary raw material is directed to landfills and constitutes a potential source of soils, ground and surface water pollution with heavy metals, fluorine, sulphates, nitrates, as well as pollution of the air with category PM_2.5_–PM_10_ particulate matter within a five-kilometre zone from the source of accumulation of the above-mentioned raw material.

Therefore, in the light of the above, there is a large-scale scientific problem that necessitates the development and implementation of a set of efficient interdisciplinary measures aimed at mitigating the anthropogenic harm to the environment. In particular, it is necessary to reduce the man-made CO_2_ emitted into the atmosphere by industrial enterprises, more efficiently recycle solid secondary raw material formed and accumulated in the dumps, as well as shift industrial production towards the manufacturing of construction materials and elements with low CO_2_, emissions, enhanced performance specifications and corrosion resistance giving longer service life. The novelty of this study involves establishing the regularities and effectiveness of forced carbonisation of nepheline slime, which is a multi-tonnage by-product of alumina production. The results of studying the structure and properties of carbonised stone from nepheline slime are also new for this type of secondary raw material. The obtained data will promote the expansion of the range of secondary raw materials capable of binding man-made CO_2_ enabling to obtain the construction products of appropriate quality and low emission of CO_2_.

## 2. Materials and Methods

### 2.1. Materials

Nepheline sludge, a large-capacity by-product of alumina production via the alkali process from the nepheline concentrates mined in the Kola Peninsula, was used as the initial raw material while performing the scientific research. For the convenience of analysis, the nepheline sludge is hereinafter referred to as the OS (original sludge). The OS sample was taken from the production facility of the Pikalevsky alumina works after sludge separation, flushing, and condensing. After all of the above stages, the OS is represented by a dispersed product with a free moisture content of 20–23% by weight. Under such conditions, the OS was placed in a tightly sealed package and delivered to the laboratory for further research. At the laboratory, the OS was dried to a constant mass at a temperature of 95 °C to exclude the possible continuation of the hydration processes for the basic mineral in the sludge. The OS retained its natural humidity (21.8%) during its delivery to the laboratory for a total of 96 h. The necessary qualitative and quantitative studies of the OS were performed after being dried. The chemical analysis of the OS was carried out through X-ray fluorescence (XRF) analysis using an Epsilon 3XLE (PANalytical) ED-spectrometer. The results of the analysis are provided in Table 1.

**Figure 1:**
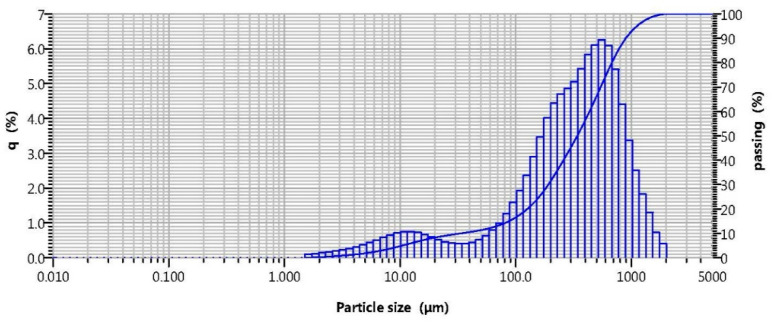
Granulometric composition of the OS particles.

The granulometric composition of the particles in the OS was determined by a Partica LA-960 (HORIBA) laser diffraction analyser. The results of the analysis are provided in Figure 1.

**Figure 2:**
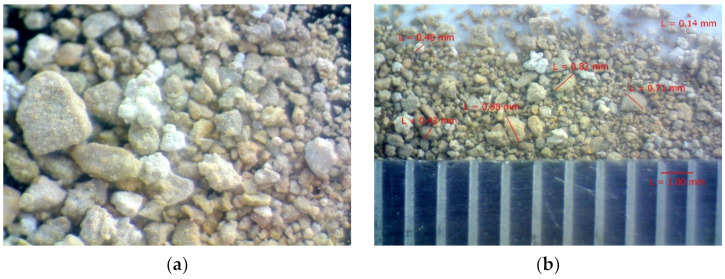
General view of the OS particles. Magnification: (**a**) ×100, (**b**) ×50.

The size of the OS particles was determined via the laser diffraction method, showing that the granulometric composition of the raw material concerned was comprised of the particles ranging from 2 to 2000 μm in size. Furthermore, the analysis of the integral and differential curves of distribution of the particles shows that the OS is represented by 2 fractions of particles, i.e., 2–50 μm and 50–2000 μm, with a quantitative ratio of 12 and 88%, respectively. The total average geometrical size of the particles within the studied sample was 250 μm. A general view of the OS particles, shapes of the granules, and size measurement with optical microscopy are provided in Figure 2.

The analysis of the OS with optical microscopy shows that the grains are angular in shape and their surface is rough. The size of the grains matches the results of the analysis of this parameter using the laser diffraction method (see Figure 1).

**Figure 3:**
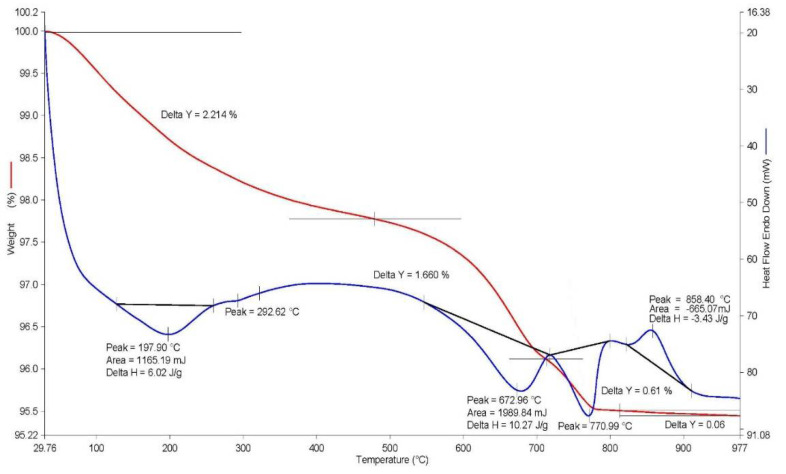
The OS thermogram.

A thermal study of the OS was performed through synchronous TG-DTA/DSC analysis executed by applying a STA 8000 (Perkin Elmer) high-temperature simultaneous thermal analyser within a temperature interval from 30 to 1000 °C at a heating rate of 20 °C/min, in a dynamic nitrogen environment. This analysis allows for the simultaneous recording of the change in heat flux and sample mass as a function of the programmed temperature at a controlled atmosphere. Such conditions allow the temperature ranges of physicochemical transformations and phase transitions to be determined with high accuracy. Calculation of the weight variation on the TG curve was performed by using the Pyris 11 (Perkin Elmer) software with the help of a DTG curve (not provided on the thermogram for the convenience of the visual analysis). The OS thermogram is presented in Figure 3.

According to the data of the thermal analysis, 3 clearly expressed endothermic effects within the temperature range of 130–800 °C, as well as one exothermic effect within the temperature range of 820–920 °C with a maximum of 858 °C are present on the DSC-curve. An insignificant endothermic effect, in terms of the area and variation of the thermal flow, with a maximum of 293 °C is also visible. The investigated sludge (OS) is a by-product of the technology of alumina production from nepheline concentrates [9]. The features of this technology theoretically allow receiving (OS) with almost monomineral composition corresponding to the stoichiometry of calcium orthosilicate (Ca_2_SiO_4_) with crystalline structure belite. Four crystalline modifications of belite (α, α’, γ and β) are known, one of which—(β-C_2_S)—manifests weak hydraulic activity and its crystallisation in the process of formation of OS is the most likely event [9]. Therefore, detailing the belonging of the analysed thermal effects to the corresponding processes of dehydration, dissociation, and phase transitions were performed based on an analysis of theoretical and practical data about the processes of formation of the alkali clinker minerals during the production of the portland cement with their subsequent hydration. It is known that β-C_2_S is the second most important mineral in standard portland cement, and the extent of its hydration over 28 days of natural hardening, as a rule, does not exceed 15% compared, e.g., with C_3_S (alite or tricalcium silicate). Correspondingly, the phase composition of the investigated OS can be represented by both the dehydrated β-C_2_S and, possibly, by the products of its hydration. In addition, the composition of OS might include sodium aluminosilicates and calcium hydrogarnets of a complex composition. The analysis of the DSC-curve confirms the theoretical data, and namely the following—the endothermic effects within the temperature range of up to 350 °C with maxima at 198 and 293 °C correspond to a partial removal of the adsorption water from the gel-like products of the β-C_2_S hydration. The endothermic effect with a maximum of 673 °C presumably corresponds to complete dehydration of the highly-alkaline calcium hydrosilicates of the type C_2_SH_2_. The endothermic effect at 771 °C with a 0.61% loss of weight corresponds to a dissociation of the calcium carbonate present within the sludge of up to 2.0% by weight. The exothermic effect within the temperature range of 820–920 °C with a maximum at 858 °C characterises the phase transition of the dehydrated calcium silicates into the wollastonite. The total loss of weight of the OS was 4.54%.

The OS crystalline phases were also analysed based on measuring the X-rays diffraction (XRD) by using a Europe 600 (GNR) diffractometer with the vertical Bragg-Brentano θ-θ-configuration equipped with a ceramic X-ray tube with a copper anode (λ = 1.541874 Å). The angular range of observation was based on 2θ was 5–80°; the scanning gauge was 0.02°, and the measuring time amounted to 35 min (2.67 degrees/minute). The total mineral composition was determined based on the powder assay of the sample, which was prepared by grinding the medium-size assay of the sample in a Retsch MM-400 oscillation mill with a milling cup and milling balls made of zirconium oxide. Identification of the compounds was performed by comparing the obtained array of reflexes with the reference diffractograms of the individual compounds by using the Match! software and the international database (ICDD PDF4 2020). Quantitative determination of the compounds was performed based on the Rietveld refinement method. The results of the XRD analysis are provided in Table 2.

On the basis of the data provided by the XRD-analysis, the investigated OS is represented primarily by β-C_2_S (belite) in the amount of 91.8% by weight, along with an insignificant number of additional minerals (see Table 2), mainly calcite in the amount of 4.5% by weight. Insignificant discrepancies in the quantitative determination of the calcite using the thermic method (2.0% by weight) can be explained by the presence of an amorphous phase in the sample, which can overestimate the content of the identified phases during the X-ray phase analysis. If this is the case, the data provided in Table 2 should be regarded as a relative estimate of the content of the identified phases that help analyse the phase variation dynamics based on the “more or less” principle.

### 2.2. Conditions for Manufacturing the Experimental Samples and the Methods for the Forced Carbonisation

Subsequent research was performed using the cylinder-shaped samples manufactured according to the method for compacting the moulding mix, which contained the OS thoroughly mixed within the high-speed mixer and the required quantity of water. The specified compositions of the moulding mix were determined by the conditions of the experiment and are presented below. The experimental samples, i.e., the cylinders having a diameter of 30 mm and a height of 30 ± 2 mm, were formed by means of compacting the prepared mixture of raw materials within the metal moulds of the hydraulic press with a simulation of two-side counter compaction. The plate-shaped samples with dimensions of 50 × 50 mm and a thickness of 8 ± 1 mm were prepared using a similar method in order to determine the bending strength.

The complex influence of technological impact on the manufacturing of the samples, as well as the mode of their forced carbonisation, was studied by using statistical methods for mathematically planning the experiment [10,11]. The rotatable central composite design (RCCD) was accepted. Given the rotatable central composite design of the experiment, the points of the design are located in three spheres—the points of the cube (N_φ_), the “star” points (N_α_), and the central points (N_o_) (the zero-radius sphere). The rotatable designs allow us to obtain the coefficients of the models that predict the values of the output total value of the object with an equivalent accuracy in all directions and in the same range from the centre of the design. The obtained experimental data were processed with the Stat Soft STATISTICA 12 software. As the result, experimental and statistical (ES) design models were made for each of the investigated parameters representing equations of the type.

(1) $$Y={\;b}_{0}+\overset{n}{\underset{i=1}{\sum }}b_{i}z_{i}+\overset{n}{\underset{i;l=1}{\sum }}b_{\mathrm{il}}z_{i}z_{l}+\sum b_{\mathrm{ii}}z_{i}^{2},$$

where b_0_, b_i_, b_il_, b_ii_ are the correlation coefficients defined as the result of the mathematical and statistical processing of the experimental data; z_i_, z_l_ are the values of the variable formulation and technological impacts.

The significance of the correlation coefficients for the ES models was determined with the help of the Student’s criterion, and verification of the correct description of the object with the help of the Fisher F-criterion.

The impacts were selected during the experiment considering the conclusions made while analysing the formation of the structure and properties of the artificial stone based on the lime of the carbonate hardening [12,13]. In the present experiment, the following were varied: the moulding pressure of the prototype cylinders (Z_1_), the initial water content of the raw mix (Z_2_), and the forced carbonisation time (Z_3_).

The conditions for planning the experiment are provided in Table 3.

The matrix used for planning the experiment in the encoded and natural expression of the variables is provided in Table 4. The value of the analysed optimisation parameter was determined as the arithmetic mean obtained while testing six experimental samples.

After the end of the forced carbonisation (see Table 4), some of the samples with residual moisture from each point of the design were placed into an ageing chamber (T = 20 °C; W = 95%) to test the samples after 28 days of natural hardening. For convenience, these samples are hereinafter designated as MH (Mixed Hardening; initially forced carbonisation + subsequent hydration). Another portion of the samples from the same point of the design was immediately dried to a constant weight at a temperature of 90 °C and subject to testing to determine the investigated parameters directly after the carbonisation. It is designated as CH (Carbonate Hardening). Therefore, the process of the hydration hardening of the material after its initial forced carbonisation was studied. Additionally, a batch of experimental samples was manufactured and then immediately placed into the ageing chamber (T = 20 °C; W = 95%) to test the samples after 28 days of natural hardening. This batch of samples was not subject to initial forced carbonisation and was designated as HH (Hydration Hardening). These samples were intended to obtain the comparison data for the pure hydration hardening (HH) and the mixed one (MH as the initially forced carbonisation + subsequent hydration).

**Figure 4:**
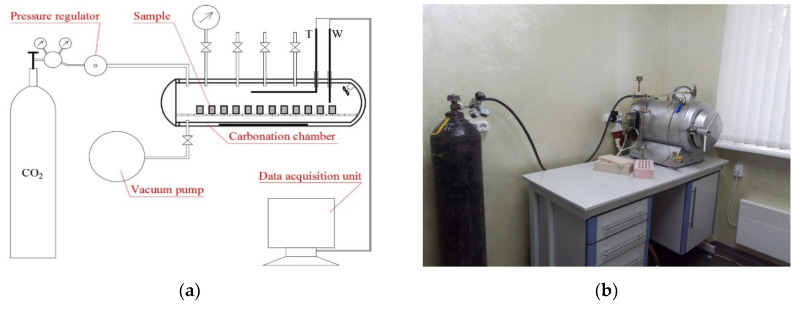
Block diagram (**a**) and general view (**b**) of the forced carbonisation chamber.

The initial forced carbonisation of the experimental samples was performed within the carbonisation facility developed by the authors offering automatic control and support of the required CO_2_ concentration [14]. This experiment was performed with the CO_2_ concentration in the chamber equal to 30% and under atmospheric pressure. The block diagram and the general view of the developed chamber are provided in Figure 4. The carbonisation chamber is represented by a metal vessel made of stainless steel with a hydraulic jacket and tightly sealed lid. The chamber is able to withstand an extensive pressure of up to 1.0 MPa. A ventilator used for stirring and uniformly distributing the carbon dioxide within the inner space was installed inside the chamber. A vacuum pump used for pumping the air out of the inner space and achieving the required concentration of CO_2_ inside the chamber was connected to the chamber. The system for the pressure control and supplying of the carbon dioxide into the carbonisation chamber included a gas cylinder, carbon dioxide, and gas pressure reducing gear.

Liquefied high-pressure carbon dioxide provided in the gas cylinders was used as the source of the carbon dioxide during the laboratory research.

### 2.3. Physical and Mechanical and Hydrophysical Properties of the Experimental Samples

According to the plan of the experiment (see Table 4), the compressive strength (R_c_), the bending strength (R_f_), the average density (ρ_o_), the water absorption by weight (W_m_), and the water resistance (K_S_) were all analysed as the basic investigated parameters of the carbonised experimental samples. The compressive and flexural strength, the average density, and the water absorption by weight were determined based on the standard methods applied to construction materials. The water-resistance of the experimental samples, i.e., the capability of the material to preserve its performance specifications under the long-term influence of water, was estimated based on the so-called K_S_ softening coefficient, represented by the ratio between the compressive strength of the sample in the saturated with water state (R_w_) to the compressive strength of the material in the dry state (R_d_) using the following formula:

K_S_ = R_w_/R_d_, (2)

The materials with a K_S_ value larger than 0.8 are considered to be water-resistant. Before the tests, all of the samples were dried in a drying cabinet to a constant weight under a temperature of 90 °C.

### 2.4. Phase Composition and Microstructure of the Experimental Samples

The phase composition of the MH and HH samples, and, correspondingly, the amount of the absorbed CO_2_, were determined with the help of thermal and radiographic phase analyses. The relevant analytical equipment and conditions for performing the experiment are described above. The porous structure of the samples, depending upon the type of hardening (MH or HH), was studied using the reference contact porometry method as the structural characteristic of the experimental samples. Variation of the porosity value was executed using Automated Standard Porosimeter 3.2 (MPM&P Research Inc., Toronto, ON, Canada). The essence of applying the method for reference contact porometry in the experiment was reduced to measuring the equilibrium dependence of the relative quantity of the measuring fluid in the investigated sample on its quantity in the standard sample, for which the porometric curve is known beforehand. For that purpose, the standard and the investigated samples were preliminarily dried, weighed, and impregnated in vacuum with the measuring fluid (octane). After removing the free fluid, the porous bodies were placed in contact with each other. Subsequently, a certain amount of the measuring fluid was removed from the above-mentioned set of porous bodies by means of evaporation. After establishing the capillary equilibrium between the porous bodies, the standards and the samples were individually weighed. The weight and volume of the fluid in the standards as well as the investigated sample were determined based on a comparison between the dry weights of the standards and the sample. Similar operations were performed until the investigated sample pores were completely free of the fluid. The sought dependence curve of the relative moisture content of the investigated sample on the value of the moisture content of the standards was determined based on the measurements performed, as mentioned above. The porometric curve was obtained for the investigated sample from the aforementioned dependence and the calibration porometric curve for the standard samples (the pore volume distribution curve upon the radii thereof). After the standards and the investigated sample were saturated with octane in the vacuum chamber and then loaded into the device, all of the subsequent drying and weighing operations were performed automatically with the help of a robotic mechanism. The obtained porometric curve was processed by specific software provided by the manufacturer of the equipment.

The microstructure of the samples was also additionally studied by using a VEGA 3 (TESCAN) scanning electronic microscope with a thermal emission tungsten cathode. The morphology of the investigated surface (spalling) was determined with the help of the obtained imaging. In order to obtain the above-mentioned images, the investigated sample was fixed on an aluminium table with double-sided conductive adhesive carbon tape. By using a Quorum Q 150T ES sputter coater, the sample was coated with a 10 nm thick layer of chromium (in order to avoid charging the samples). The fragments of the material (MH) obtained after the compression test of the cylinder-shaped samples were used as the investigated samples.

## 3. Theory

A steady growth in the number of studies on climate change, environmental problems, and enhancing the service life of the materials [15,16,17]—especially in the manufacturing and construction sectors–has been observed over the last decade. This is because most of the above-mentioned products are manufactured based on portland cement, the production of which is associated with significant emissions of anthropogenic CO_2_ into the atmosphere. The majority of the papers in this respect are related to studying how to reduce the CO_2_ emissions while manufacturing portland cement, its hardening under the influence of CO_2,_ and the technological impacts influencing the process concerned. However, studies have shown that an average of 0.9–1.1 tonnes of CO_2_ per tonne of portland cement is produced, and the CO_2_ binding potential of the cement itself does not exceed 5% of the cement mass. This indicator varies significantly depending on the mineralogical composition of the portland cement clinker [18,19].

Correspondingly, the construction materials based on the cement cannot make any substantial contribution to decreasing global emissions of CO_2_. In contrast, the investigations show that the types of cement with a higher content of belite bind more carbon dioxide and thus obtain stronger physical and mechanical characteristics as compared with alite cements. This observation has driven scientists to search for alternative solutions applied to the partial or complete substitution of portland cement in the manufacturing of construction materials [20,21]. The result of the above-mentioned research was represented by studying the possibility of the carbonate hardening of the metallurgic blast furnace slags containing 60–65% belite (C_2_S). It is known that even when finely ground, such slags possess no hydraulic activity and alkali components are required to activate it for subsequent hardening. Research has shown that this slag has a high reactivity for forced carbonisation (carbonate hardening). The carbonisation products (calcium/magnesium carbonates, calcium silicate hydrates) are the main phases that have binding properties and are responsible for the formation of the performance characteristics of the resulting material [22,23].

It was not that after the process of artificially carbonising the systems containing C_2_S, total porosity substantially decreased and a significant amount of the closed pores originates. It was also detected that artificial carbonisation of the blast furnace slags facilitates the formation of both binding porosity within the experimental samples, and the closed variety, as the result of which the properties of the carbonised stone vary substantially. The formation of various kinds of porosity depends primarily on the ratio of the polymorphous modifications of C_2_S in the slag and the conditions for running the process of forced carbonisation. In quantitative terms, the total porosity value decreases by 34 to 76%, depending upon the period of carbonisation. The number of macropores falls in this case, whereas the number of micropores and mesopores rises due to the formation of calcium and magnesium carbonate crystals at the nano-dimensional level [24,25,26,27,28,29,30,31,32,33,34,35,36]. In this case, the nepheline sludge (a by-product formed during the production of alumina with the help of alkali process from the nepheline concentrates mined in the Kola Peninsula) containing up to 95% belite, is still a more promising secondary raw material for the production of construction materials with low CO_2_ emissions. Specific scientific research on carbonate hardening of a given secondary raw material was not mentioned in scientific references.

Attention should also be paid to a company named Carbon8 Systems [37] founded in Great Britain based on the University of Greenwich (London, UK) in 2006. The company is a result of the commercialisation of scientific ideas in the sphere of forced carbonisation of anthropogenic waste. Professor Colin Hills, a co-founder of Carbon8 Systems, was the instigator of this research trend. Thus, the first experiments in the production of artificially carbonised fillers for types of concrete based on the ash from thermal power plants and cement dust settling in the duct bag filters of cement works were made in the city of Brandon, the United Kingdom as early as in 2012. Two more similar endeavours were conducted in Canada and the USA in 2016. The productive capacity of the above-mentioned enterprises amounts up to 120 thousand tons of solid anthropogenic waste capable of binding up to 30 thousand tons of gaseous CO_2_. As a result, an artificial porous filler was produced (with a bulk density of up to 1 200 kg/m^3^), which is on a par with the traditionally used ceramsite gravel. In 2017, the company received the Queen’s Award for Enterprise for “Innovation 2017”. The technological solution that captures CO_2_ directly from the smoke-stack gases from the kilns of the Hanson cement works in the United Kingdom (a member of Haidelberg Cement Group) was put into operation in 2019. Presently, Carbon8 Systems remains the only company offering its own in-house developed technological solutions in the sphere of forced carbonisation in relation to the manufacturing of the construction materials on a commercial basis; and the research and development department of the company is actively engaged in studying the forced carbonisation of blast furnace slags taken from European ferrous metal enterprises.

Therefore, due to the above, the studies related to the possibility of forced carbonisation of nepheline sludge and obtaining construction materials with high-performance specifications are both relevant and modern.

## 4. Results and Discussion

### 4.1. Influence of Varying Process Factors on the Performance of Prototypes

Experimental data of the properties of the experimental samples, according to the plan of the experiment, are provided in Table 5.

The calculated correlation coefficients for the ES-models obtained as the result of the statistical processing of the experimental data are provided in Table 6.

The analysis of the correlation coefficients for the ES-models, while considering various combinations of technological impacts influencing the formation of the structure and the properties of the investigated experimental samples of the carbonate hardening, allow us to conduct an analysis on the impacts and their interaction, determining every single property at the technological stage taken separately. On the basis of the variation of the correlation coefficients at different stages of the technology, the role and the degree of the influence exerted by different impacts upon the formation of the properties of the experimental samples, can be judged depending on the composition of the moulding mix, the conditions for manufacturing the samples and their forced carbonisation.

The experimental data and the correlation coefficients for the ES-model prove the variation of the basic investigated properties of the carbonised samples after their ageing over 28 days. While estimating the coefficient b_o_, it can be stated that only the compressive strength (R_c_) and the water resistance (K_s_) vary essentially after 28 days of hardening, while the variation in the other properties is insignificant. Furthermore, it should be noted that the density (ρ_o_) of the carbonised samples decreases after 28 days of hardening, and the water absorption (W_m_) increases.

Estimation of the technological impacts taken separately indicates that the importance of their influence upon the formation of the properties of the materials based on the carbonised OS is different. Thus, if the initial water content of the mixture (Z_2_) (model 1, b_2_ = −18.02) exerts a determining influence upon the compressive strength (R_c_) immediately after the carbonisation, the duration of the carbonisation (Z_3_) (model 2, b_3_ = 14.81) becomes the determining factor for this parameter after 28 days of hardening. However, it is noteworthy that the difference in the absolute values between the coefficients b_2_ and b_3_ (−18.02 and 17.47 correspondingly) is not very large immediately after the carbonisation (model 1), which indicates a substantial bilateral influence of the impacts (Z_2_) and (Z_3_) upon the formation of the properties of the carbonised samples. A similar situation is also observed while analysing the coefficients applied to other investigated properties (R_f_, ρ_o_, W_m_, K_s_) of the experimental samples. Thus, if the compaction pressure (Z_1_) has the most significant impact on the bending strength and the water resistance (R_f_ and K_s_) immediately after the carbonisation, then the duration of the carbonisation (Z_3_) becomes a significant influence on the density and water absorption (ρ_o_ and W_m_). Generalised analysis of the coefficients (see Table 6) shows that all of the selected technological impacts (see Table 3) substantially influence the qualitative and quantitative course of the chemical carbonisation reaction of the basic OS mineral (β-C_2_S), its subsequent hydration hardening, and, correspondingly, the formation of the properties of the obtained material as well. It is also worth mentioning that the impacts which are directly responsible for the process of the running of the chemical reaction of carbonisation include the initial water content of the mix (Z_2_) and the duration of carbonisation (Z_3_).

In order to obtain the comparison data concerning the process of the pure hydration hardening of the investigated OS, a batch of the experimental samples was additionally manufactured and then immediately placed into the ageing chamber (T = 20 °C; W = 95%) to test the samples after 28 days of natural hardening. Correspondingly, this batch of the experimental samples was not subject to the forced carbonisation. The manufacturing conditions of the above-mentioned samples complied with the conditions of the central points of the plan of the experiment (see Table 4). The results of the tests are provided in Table 7.

The analysis of the data provided in Table 7 shows that the investigated OS possesses weak hydraulic activity, which is revealed in an insignificant increase in the compressive strength and the bending strength by the age of 28 days. Decreasing the average density value by 28 days of hardening also confirms the running of the processes of hydration of belite (β-C_2_S) accompanied by the formation of the capillary-porous structure of the material, and, correspondingly, by decreasing its density. It is noteworthy that for the samples (HH) the compressive strength at 28 days of hardening amounted to 13.0 MPa, whereas the similar parameter of the preliminary carbonised samples (see Table 5) varied within the limits of 24.66–68.71 MPa (points No. 13 to 20) and depended on the duration of the carbonisation. At the same time, already after 19 min of the forced carbonisation (see Table 5, point No. 13) the compressive strength of the samples comprised 11.23 MPa, which was practically comparable with the given parameter (13.0 MPa) for the samples not subject to carbonisation and that had hardened during the 28 days. In general, the analysis of the data (Table 5 and Table 7) shows that the samples (MH) (Table 5) possess much higher and far more preferable parameters of their physical and mechanical, and hydrophysical properties, compared to similar parameters obtained after pure hydration hardening (HH) (Table 7).

### 4.2. Phase Composition of the Samples Depending upon the Mode of Their Hardening

**Figure 5:**
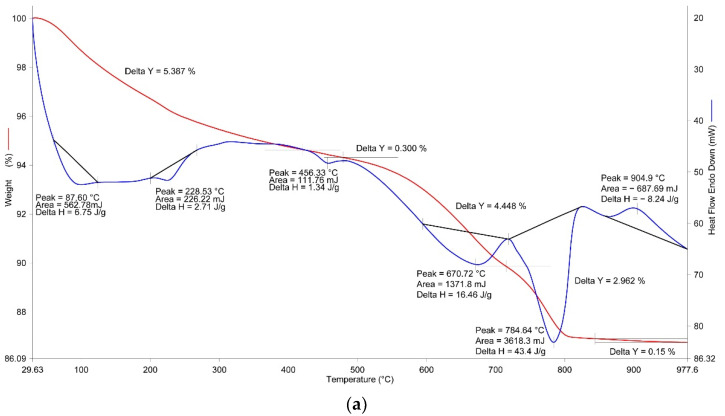

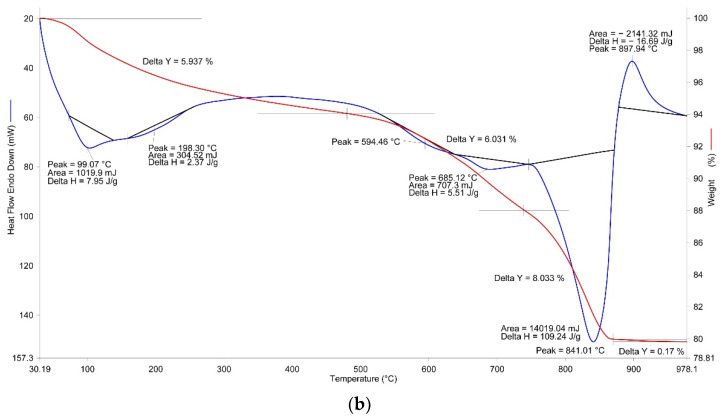
Results of the TG-DSC analysis of the HH (**a**) and MH (**b**) samples.

In addition to the variation of the properties of the material after the carbonisation, the reaction efficiency of the carbonisation process is also related to the quantitative absorption of the carbon dioxide per unit of weight of the material concerned. This parameter is a determining factor in terms of understanding the perspective of applying another material capable of binding CO_2_ and, thus, contributing [4] to reducing the emission of this greenhouse gas into the atmosphere. Thermal and radiographic phase analyses methods were used in the conducted research to determine the quantity of CO_2_. According to the plan of the performed experiment, the point of design No. 14 (see Table 5) was characterised by the highest increment in the weight of the samples after carbonisation under various combinations of technological impacts for manufacturing the samples. Therefore, the thermal and radiographic phase analyses were performed for the given samples, as well as for those not subject to carbonisation and hardened under the hydration mechanism only (Table 7) after 28 days of hardening. The data of the thermal analysis are represented in Figure 5.

According to the thermal analysis data, the configuration of the curves and thermal analysis characteristics of the investigated assays are slightly different. Therefore, the TG and DSC curves of the thermogram (Figure 5a) repeat the configuration of similar curves of the OS (see Figure 3). The difference is represented by an increased loss of weight on the TG curve within the relevant temperature intervals and by increasing the area of the thermal effects while preserving the temperature maximums as confirmed by the occurrence of the process of hydration of belite (β-C_2_S) and formation of a more essential quantity in the gel-like products of hydration. The occurrence of the endothermic effect within an interval of 420–479 °C with the maximum at 456 °C can be also regarded as a factor which confirms that the hydration processes occur with the appearance of additional reaction products. This effect corresponds to dehydration of Ca(OH)_2_. The quantity of Ca(OH)_2_ was calculated based on the value of loss of weight consisting of 1.23%. It should be noted that variation of the weight of the assay within a range of 820–977 °C related to the phase transition of the dehydrated calcium silicates into wollastonite amounted to 0.15%—that is, 2.5 times more than the similar parameter (0.06%) for the OS (see Figure 3). The total loss of weight amounted to 13.25%.

**Figure 6:**
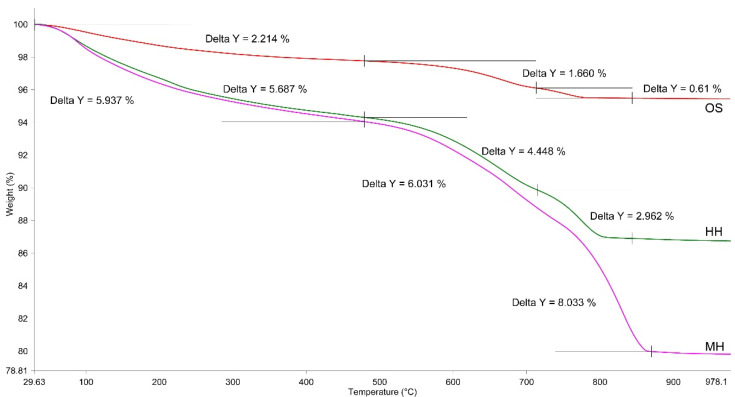
Results of the TG analysis of the OS, HH, and MH samples.

The DSC curve of the carbonised samples subsequently hardened within 28 days (Figure 5b) has 2 distinctive features compared to the similar curve (Figure 5a). Thus, the endothermic effect characterising calcium carbonate availability has a substantially larger surface area and essentially shifts towards a higher temperature maximum—namely 841 °C, as compared with 784.6 °C for the uncarbonised samples. The difference in the loss of weight characterising the quantity of CO_2_ released is also relevant and amounts to 8.0 and 2.9%, respectively. A more detailed analysis of variation of the weights of the investigated samples within the relevant temperature ranges is provided in Figure 6 and Table 8.

An estimation of the quantitative parameters of variation in the weight of the investigated samples (see Figure 6, Table 8) shows that the preliminary carbonisation of the sludge facilitates a greater extent of hydration of belite (β-C_2_S) after 28 days, as compared to a similar sample which was not subjected to initial carbonisation. Thus, the extent of hydration of the samples hardened under the hydration and mixed types comprised 9.84 and 11.97%, respectively. The quantity of CO_2_ was also larger—amounting to 2.96 and 8.03%, respectively, and the content of CaCO_3_ calculated based on the above values was 6.7 and 18.3%. Therefore, based on the aforementioned conditions for the performance of the experiment, 1 ton of the studied nepheline sludge is capable of binding 80.3 kg of CO_2_, simultaneously obtaining a certain specification of additional construction products with high-performance characteristics. It should be mentioned that for the samples hardened under the mixed mode, a shift in the temperature range towards higher temperatures was observed similar to the processes of complete dehydration of the highly-alkaline calcium hydrosilicates and the dissociation of CaCO_3_ that proves a higher content of such compounds in the assay and their enhanced extent of crystallinity.

**Figure 7:**
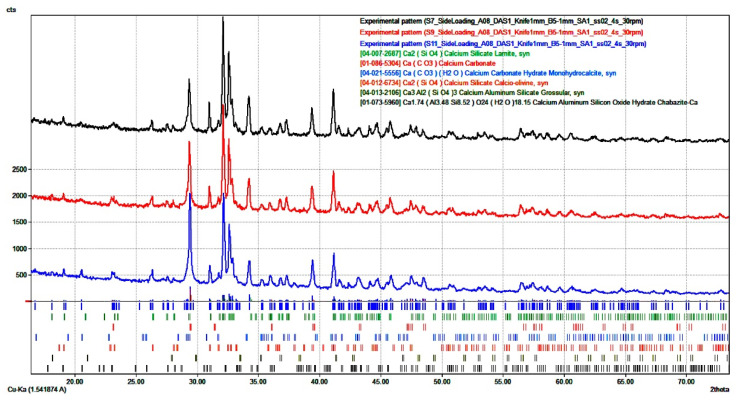
Results of the XRD analysis of the OS, HH, and MH samples.

The thermal analysis data are fairly well correlated with the data of the radiographic phase analysis, the results of which are provided in Figure 7 and Table 9.

The analysis of the data (see Table 9), along with a similar analysis for the OS (see Table 2), shows that this raw material features weak hydraulic activity when hardened under the hydration mode (HH) only. This is expressed in an insignificant decrease in the β-C_2_S quantity after 28 days of hardening (88.6%) as compared with the similar parameter for the OS (91.8%), as well as in low values of compressive and flexural strength (see Table 7). The quantity of β-C_2_S for the samples subject to the initially forced carbonisation and further hardened within 28 days (MH) drops to 71.3%. This proves that the said mineral chemically reacts with CO_2_ and forms calcium carbonate—possessing the binding properties and facilitating an essential increase in physical and mechanical properties of the carbonised material—as the product of the reaction. In this case, β-C_2_S, which has not initially reacted with the forced carbonisation, continues to be hydrated more intensely with further ageing of the samples, thus indicating greater hydration and a more substantial increase in the mechanical properties of the carbonised material, as compared with the pure hydration hardening. Reducing the intensity of the baselines for β-C_2_S is observed in the roentgenogram (see Figure 7); and correspondingly—an increase along similar lines for the calcium carbonate, especially for the samples, which are hardened under the mixed hardening (MH) mode. Thus, the quantity of CaCO_3_ for the HH and MH samples reached 7.4 and 20.8%, respectively. Slightly larger quantity of the calcium carbonate, as compared to the data of the thermal analysis (6.7 and 18.3%), can be explained by a probable availability in the assay of the amorphous phase, which can ensure higher quantitative parameters while performing the XRD analysis.

### 4.3. Structure of the Samples Depending on the Mode of Their Hardening

**Figure 8:**
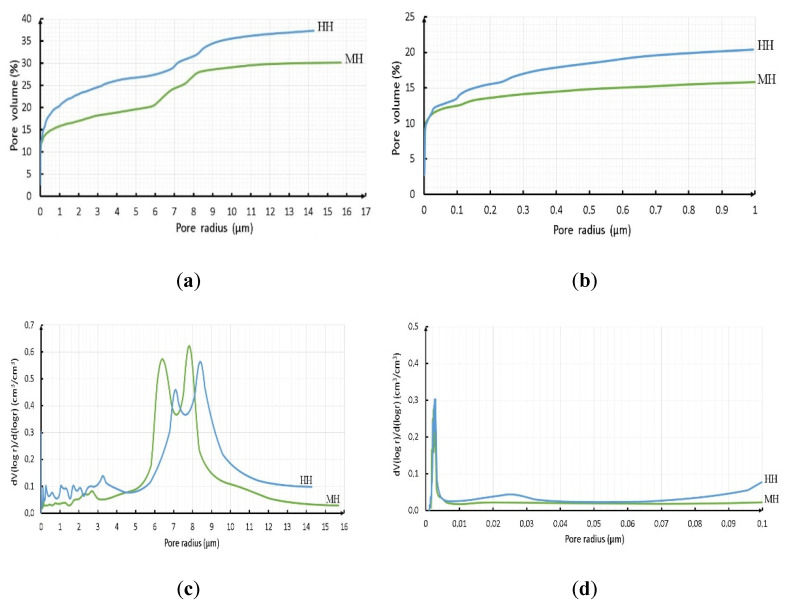
Integral (**a**,**b**) and differential (**c**,**d**) characteristics of the porous structure of the investigated HH and MH samples.

The analysis of the porous structure of the HH and MH was performed with the help of reference contact porometry method. The results of the analysis are provided in Figure 8 and Table 10.

On the basis of the obtained results, a conclusion can be drawn that the mode of the porous structure of the material is formed in the process of compaction of the samples and depends on the percentage of the fractional composition of the initial raw material; moreover, the initial carbonisation makes an essential contribution to decreasing the total porosity of the material. The total porosity of the MH samples after 28 days of hardening is decreased by 7.5%, compared to the HH samples. Furthermore, a detailed analysis of the integral and differential curves shows that substantial differences in the porosity values of the investigated samples begin at an effective pore radius value of more than 0.1 μm. At a pore radius of less than 0.1 μm the curves are practically superimposed on each other, meaning that the reaction of carbonisation has not taken place within those pores. The number of pores in a given radius was practically the same for the 2 samples—from 12.7 to 13.5%. The analysis of the differential curves (see Figure 8c,d) shows that within the range of 6–9 μm the pore radii of the MH samples shifted towards smaller values, as compared with the HH samples. This can be explained by the accumulation of the products of carbonisation and subsequent hydration on the pore walls, as the result of which the pore radius decreases, and larger pores split into smaller ones.

It should be noted that the total volume of the pores within the range of the radii from 0.1 to 15 μm amounted to 14.3 and 24.0% for the MH and HH samples, respectively. These values correlate rather accurately with the experimental data in relation to the water absorption by weight during 28 days for the given samples (14.2 and 22.2%). Thus, it can be assumed that the porosity with a pore radius less than 0.1 μm is closed and, correspondingly, is inaccessible to water. Therefore, the MH type samples are far more preferable both from the point of view of improved properties and the quantitative absorption of CO_2_. Additional research on the material microstructure with the help of scanning electron microscopy was performed for the above-mentioned samples.

**Figure 9:**
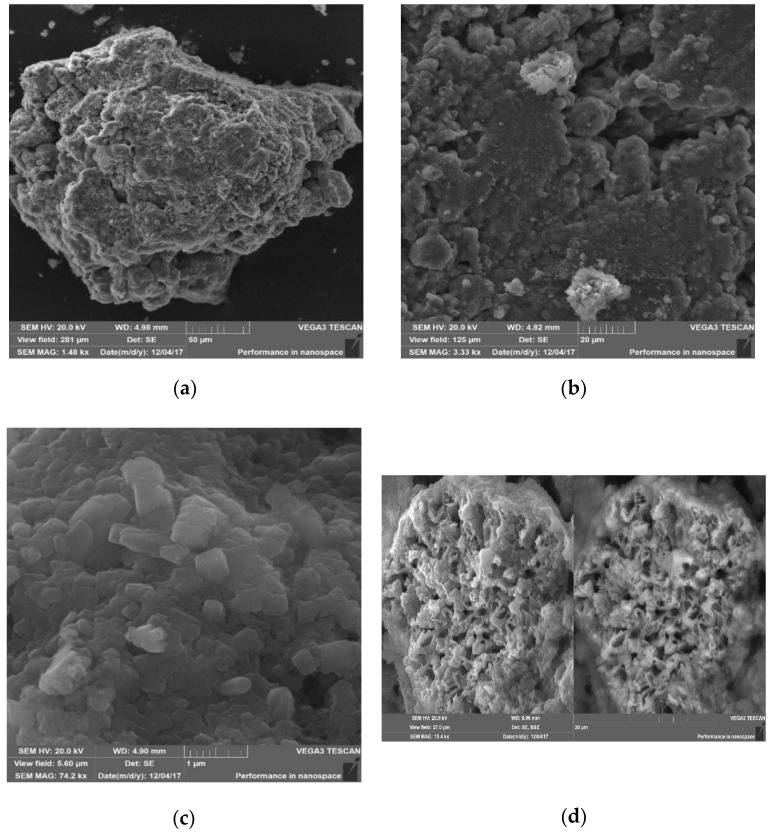
Results of studying the microstructure of the carbonised samples: (**a**,**b**)—general view; (**c**)—CaCO_3_ crystals; (**d**)—products of the β-C_2_S hydration.

The structure of the MH samples is provided in Figure 9. It is evident from the provided images that the porous structure of the material is represented by both open and closed pores (see Figure 9a,b), whereas the number of closed pores increases while the pore sizes decrease. The CaCO_3_ scalenohedral crystals (see Figure 9c) with dimensions ranging from 0.1 to 1.0 μm, as well as presumably filamentary (gel-like) products of the β-C_2_S hydration (see Figure 9d) located within the porous space of the material are present as the carbonisation reaction products. Generally speaking, it should be mentioned that the SEM data match the data obtained while studying the porous structure of the MH samples using the reference contact porometry method.

## 5. Conclusions

An analytical scientific review of the disposal of production waste and, in particular, of solid nepheline sludge and gaseous carbon dioxide determined that the solid waste is primarily accumulated in the dumps of industrial enterprises (nepheline sludge) and emitted into the atmosphere (as carbon dioxide). The obtained experimental results lead to the conclusion that the potential of such kinds of raw materials is not used to the full extent in the manufacturing of construction materials and elements—in particular, towards the development of materials with low CO_2_ emissions. The optimisation of the technological parameters for obtaining the carbonised material with high-performance specifications based on the nepheline sludge was performed with the help of the method for mathematical planning of the experiment. Carbonised material was obtained with compressive strength within the limits of 4.38–68.71 MPa, and flexural strength of 3.1–8.9 MPa. The value of the water absorption by weight is within the limits of 13.9–23.3%, and the average density—between 1640 and 1886 kg/m^3^. The softening coefficient of the material was equal to 0.51–0.99. A detailed analysis of the experimental data determined that the initially forced carbonisation of the experimental samples facilitates the occurrence of CaCO_3_ that has binding properties within the structure of the material, while also intensifying the subsequent hydration process of β-C_2_S, which is not engaged in the carbonisation reaction, with the formation of additional gel-like products of hydration that enhance the performance specifications of the end material. The extent of hydration of the material is also boosted compared to the samples of the hydration type of hardening only. It was determined that when using the investigated sludge in its natural granulometric state, the quantity of the bound CO_2_ per one ton of original sludge amounted to 80.3 kg at given technological impacts (see Table 5). Theoretically, this parameter can essentially be increased with the introduction of additional technological impacts and the methods for the preparation of the initial OS into the research data. A generalised analysis of the obtained results assumes that manufacturing a certain specification of construction elements with the carbonate type of hardening, including elements of paving, which would meet the requirements set out by the current regulatory documents and technical standards, is possible with the given type of raw material via the suggested method for initially forced carbonisation, and use of a secondary raw material (the nepheline sludge and CO_2_ emissions) and the fast-action process cycle of the production would correspondingly decrease the production costs of the ready-made carbonised construction elements.

## Figures and Tables

**Table 1:** Chemical composition of the OS.

Name	Content if Recalculated as Oxides (%)							
	CaO	MgO	SiO_2_	Al_2_O_3_	Fe_2_O_3_	Na_2_O	K_2_O	Total (%)
**OS**	63.41	0.72	27.73	3.24	2.51	1.41	0.91	99.93

**Table 2:** Identification of the OS crystalline phases and estimation of their quantitative content.

Phase	Formula	Weight (%)
Belite-β (Larnite)	Ca_2_SiO_4_	91.8
Belite-α’	Ca_2_SiO_4_	2.0
Calcite	CaCO_3_	4.5
Monohydrocalcite	CaCO_3_·H_2_O	1.4
Grossular	Ca_3_Al_2_(SiO_4_)_3_	0.2
Chabazite	Ca_1.74_ (Al_3.48_ Si_8.52_) O_24_·(H_2_O)_18.15_	0.1

**Table 3:** The conditions for planning the experiment.

Description of the Impact	Unit of Measure	Code	Variation Levels					Variation Interval
			−1.682	−1	0	1	1.682	
Compaction pressure	MPa	Z_1_	13.18	20.0	30.0	40.0	46.82	10.0
The water content of the mix	%	Z_2_	6.59	10.0	15.0	20.0	23.41	5.0
Duration of the carbonisation	min	Z_3_	19.08	60.0	120.0	180.0	220.92	60.0

**Table 4:** The matrix for the planning of the experiment in the encoded and natural expression of the variables.

No.	The Plan of the Experiment						
	Groups of the Points	Planning Matrix			Natural Values of the Variables		
		Z_1_	Z_2_	Z_3_	Compaction Pressure of the Mix (MPa)	The Water Content of the Mix (%)	Duration of the Forced Carbonisation (Min)
1	N_φ_ (cube points)	−1	−1	−1	20.00	10.00	60.00
2		1	−1	−1	40.00	10.00	60.00
3		−1	1	−1	20.00	20.00	60.00
4		1	1	−1	40.00	20.00	60.00
5		−1	−1	1	20.00	10.00	180.00
6		1	−1	1	40.00	10.00	180.00
7		−1	1	1	20.00	20.00	180.00
8		1	1	1	40.00	20.00	180.00
9	Nα (star points)	−1.682	0	0	13.18	15.00	120.00
10		1.682	0	0	46.82	15.00	120.00
11		0	−1.682	0	30.00	6.59	120.00
12		0	1.682	0	30.00	23.41	120.00
13		0	0	−1.682	30.00	15.00	19.08
14		0	0	1.682	30.00	15.00	220.92
15	No (center points)	0	0	0	30.00	15.00	120.00
16		0	0	0	30.00	15.00	120.00
17		0	0	0	30.00	15.00	120.00
18		0	0	0	30.00	15.00	120.00
19		0	0	0	30.00	15.00	120.00
20		0	0	0	30.00	15.00	120.00

**Table 5:** Experimental data of the properties of the experimental samples were obtained immediately after the carbonisation (CH) and after a subsequent 28 days of hardening (MH).

No.	Variable Factors in the Natural Expression			Optimized Parameters									
	Z_1_ (MPa)	Z_2_ (%)	Z_3_ (Min)	R_c_ (MPa)		R_f_ (MPa)		ρ_o_ (kg/m^3^)		W_m_ (%)		K_s_	
				CH	MH	CH	MH	CH	MH	CH	MH	CH	MH
1	20.00	10.00	60.00	16.84	22.86	5.1	6.9	1712	1713	20.0	19.3	0.97	0.85
2	40.00	10.00	60.00	43.62	46.27	7.9	8.4	1835	1844	17.4	16.8	0.82	0.85
3	20.00	20.00	60.00	5.44	22.95	4.4	4.6	1640	1674	23.3	19.7	0.97	0.84
4	40.00	20.00	60.00	6.37	26.18	3.1	4.9	1747	1745	20.2	19.7	0.76	0.62
5	20.00	10.00	180.00	23.22	24.05	4.3	4.3	1828	1812	20.5	19.0	0.82	0.81
6	40.00	10.00	180.00	44.72	43.53	8.2	8.2	1997	1851	16.7	16.2	0.89	0.86
7	20.00	20.00	180.00	14.21	44.65	7.5	8.9	1670	1741	18.5	17.1	0.99	0.91
8	40.00	20.00	180.00	10.81	25.59	6.7	7.4	1765	1792	18.6	17.5	0.78	0.88
9	13.18	15.00	120.00	29.21	25.91	3.5	3.3	1640	1645	20.9	19.5	0.72	0.86
10	46.82	15.00	120.00	57.64	59.18	7.0	7.2	1870	1882	15.9	15.0	0.79	0.84
11	30.00	6.59	120.00	23.08	28.74	4.0	4.5	1760	1764	18.2	18.6	0.88	0.77
12	30.00	23.41	120.00	4.38	15.79	3.6	3.8	1706	1713	22.7	20.4	0.80	0.68
13	30.00	15.00	19.08	11.23	24.66	3.1	3.7	1709	1700	20.7	21.5	0.98	0.51
14	30.00	15.00	220.92	54.94	68.71	6.4	7.8	1886	1865	13.9	14.2	0.90	0.75
15	30.00	15.00	120.00	26.55	47.00	5.0	7.2	1810	1770	15.1	17.3	0.94	0.77
16	30.00	15.00	120.00	23.31	45.32	4.9	7.1	1791	1741	15.9	18.3	0.94	0.73
17	30.00	15.00	120.00	24.00	45.08	4.9	7.0	1795	1757	15.8	17.5	0.91	0.75
18	30.00	15.00	120.00	26.11	46.23	5.1	7.1	1807	1761	15.3	16.7	0.93	0.77
19	30.00	15.00	120.00	25.57	44.08	5.2	6.9	1802	1758	15.1	17.0	0.94	0.76
20	30.00	15.00	120.00	24.78	46.63	5.5	7.2	1797	1746	15.3	16.8	0.91	0.78

**Table 6:** The correlation coefficients for the ES-models of variation of the basic properties of the experimental samples were obtained immediately after the carbonisation (CH) and after a subsequent 28 days of hardening (MH).

Description of the Coefficients	Coefficients for the ES-Models of the Investigated Parameters Depending upon the Duration of the Test									
	R_c_ (MPa)		R_f_ (MPa)		ρ_o_ (kg/m^3^)		W_m_ (%)		K_s_	
	CH	MH	CH	MH	CH	MH	CH	MH	CH	MH
b_o_	25.87	46.6	4.82	6.98	1799.1	1754.6	15.45	17.81	0.94	0.74
b_1_	10.03	11.06	1.77	1.55	104.4	90.6	−2.19	−1.94	0.11	0.039
b_2_	−18.02	−5.73	−0.65	−0.465	−93.8	−51.8	1.99	0.84	−0.02	−0.039
b_3_	17.47	14.81	1.48	1.62	115.9	83.4	−3.05	−2.52	−0.09	0.029
b_11_	8.54	−4.7	0.83	−0.515	−25.3	10.1	2.28	−0.42	−0.011	0.114
b_22_	−12.45	−19.04	−0.197	−1.29	−40.9	−7.5	3.73	1.17	−0.054	0.026
b_33_	1.23	−1.78	0.474	−0.16	4.76	23.6	1.50	0.01	0.016	−0.041
b_12_	1.43	5.66	1.80	2.40	−57.5	2.0	−1.55	−0.98	0.03	0.090
b_13_	−2.40	−6.56	0.40	0.15	8.5	−28.0	0.50	0.03	0.055	0.060
b_23_	−12.69	−14.68	−2.20	−1.65	−22.5	−12.0	0.85	1.43	0.085	−0.075

**Table 7:** Experimental data of the properties of the experimental samples (HH) not subject to the initially forced carbonisation and hardened within 28 days.

No.	Manufacturing Conditions		Properties of the Samples (HH) Depending upon the Period of Hardening (Days)																			
	Z_1_ (MPa)	Z_2_ (%)	3	7	14	28	3	7	14	28	3	7	14	28	3	7	14	28	3	7	14	28
			R_c_ (MPa)				R_f_ (MPa)				ρ_o_ (kg/m^3^)				W_m_ (%)				K_s_			
1	30.00	15.00	3.3	4.7	9.5	13.0	0.5	0.9	1.6	1.9	1739	1737	1707	1701	24.3	24.1	23.4	22.2	0.91	0.91	0.94	0.95

**Table 8:** Detailed analysis of variation of the weight of the investigated samples within the relevant temperature ranges depending on the nepheline sludge hardening conditions.

Description	Temperature Range (°C)					Total Loss of Weight (%)	
	30–479	479–715	479–740	715–820	740–880		
	Loss of Weight (%)					H_2_O	CO_2_
OS	2.21	1.66	-	0.61	-	3.87	0.61
HH	5.39/0.3 *	4.45	-	2.96	-	9.84	2.96
MH	5.94	-	6.03	-	8.03	11.97	8.03
Physical and chemical variations	partial removal of the adsorption water from the gel-like products of the β-C_2_S hydration	complete dehydration of the highly-alkaline calcium silicates		dissociation of CaCO_3_			

Note: 5.39/0.3 *—0.3% corresponds to dehydration of Ca(OH)_2_ (see Figure 5a) and is not considered while calculating the extent of hydration.

**Table 9:** Identification of the crystalline phases of the samples and estimation of their quantitative content.

Phase	Formula	HH	MH
		Weight (%)	
Belite-β (Larnite)	Ca_2_SiO_4_	88.6	71.3
Belite-α’	Ca_2_SiO_4_	1.3	1.9
Calcite	CaCO_3_	7.4	20.8
Monohydrocalcite	CaCO_3_·H_2_O	1.4	5.2
Grossular	Ca_3_Al_2_(SiO_4_)_3_	0.6	0.6
Chabazite	Ca_1.74_ (Al_3.48_ Si_8.52_) O_24_·(H_2_O)_18.15_	0.3	0.2

**Table 10:** Quantitative distribution of pores in the samples (%) depending upon their radii and the mode of hardening.

Description	Pore Radius (μm)							Total (%)
	0–0.1	0.1–1.0	1.0–6.0	6.0–7.0	7.0–8.0	8.0–9.0	9.0–15.0	
	Pore Volume (%)							
HH	13.5	7.0	7.0	2.0	2.0	3.0	3.0	37.5
MH	12.7	3.3	4.0	4.5	3.0	1.0	1.5	30.0

## Data Availability

No new data were created or analyzed in this study. Data sharing is not applicable to this article.
